# Miscellaneous

**Published:** 1887-07

**Authors:** 


### MISCELLANEOUS.

Chinese Philosophy—In ancient times there lived a man named Shien. During a traveling tour he had occasion to rest the night at a road house. The weather was insufferably hot, and within the room mosquitoes swarmed by thousands. Shien fortunately had provided himself with curtains, but unfortunately the curtains were insufficient to resist the enemy. His efforts to keep them out were in vain, sounds of buzzing in unpleasant proximity still continued, and writhing under the intolerable torment of their stings, his thoughts transplanted themselves to his own peaceful home. He reflected on the spacious halls, cool couches, and the crowd of handmaids to fan and wait on their lord ; and, continued he to himself, "how is it that I should have suffered one moment of ennui in such a paradise? Why leave to seek pleasures and find misery abroad ?" During these meditations he observed the keeper of the post, who had no curtains, pacing the room with the mosquitoes swarming around him. But what seemed to him inexplicable, was that the man still appeared to be in perfect good humor. Shien, still writhing in misery, exclaimed : "My good fellow, you are one hundred times worse off than myself, but why is it that, while I am in torment of mind, you on the contrary, seem happy ?" The keeper replied : "Sir, I have just been recalling to mind the position I was once placed in when a prisoner : bound hand and foot, I was a helpless prey to these murderous insects ; unable to move a muscle, they preyed on me with impunity, and the agony was unbearable. It was the contrast of that horrible period with my present condition that produced that feeling of contentedness with me." Shien was startled by the mine of philosophy herein unfolded. Would, he thought, that the world in ordinary life would but daily keep in mind, and carry out, such a spirit of analogy. How vast then would be the result to man !—*North China Herald.*

Pure Air at Night.—The season of the year is approaching in which doors and windows are usually closed, and the matter of pure air becomes one of serious importance. During the day, the air of living-rooms is pretty certain to be changed more or less by the frequent opening of outside doors. During the night, however, not infrequently all outside openings are tightly closed, and the occupants of sleeping-rooms might almost as well place themselves for the eight or ten sleeping hours of night in an air-tight box.

In the morning, persons who thus deprive themselves of life-giving oxygen, the great necessity of life, awake unrefreshed and dispirited, languid, pale and weak, with headache, giddiness, no appetite, and many other symptoms of foul air poisoning, to which the system has been subjected. This accounts for a very large part of the colds and other forms of physical wretchedness, of which a good many complain at this season of the year, and which is ordinarily ascribed to the change of season. The system is filled with impurities as a result of deficient oxygenation of the blood, and so the body becomes, in a high degree, susceptible to all causes of vital disturbance. The reception of a few fever germs is all-sufficient to bring on a violent illness, by setting fire to the fever breeding material with which the tissues are filled, as the result of deficient air cleansing.

Ventilation of living rooms is of great importance at all times, but the supply of an ample amount of fresh air to sleeping-rooms, is doubly important during the hours of sleep.—*Good Health.*

Disorder of Digestion.—All the vital functions are more or less processes of combustion, and they are subject to laws similar to those which regulate the burning of coal in our fireplaces. The reason why we allow our fires to burn low or go out altogether, is that we put on too much coal or that we allow them to be smothered in ashes. It is the child who pokes the fire from the top to break the coal and make it burn faster : the wise man pokes it from below, so as to rake out the ashes and allow free access of oxygen. And so it is with the functions of life, only that these being less understood, many a man acts in regard to them as a child does to the fire. The man thinks that his brain is not acting because he has not supplied it with sufficient food. He takes meat three times a day, and beef tea, to supply its wants, as he thinks, and puts in a poker to stir it up in the shape of a glass of sherry or a nip from the brandy bottle. And yet, all the time, what his brain is suffering from is not lack of fuel, but accumulation of ash, and the more he continues to cram himself with food and to supply himself with stimulants, although they may help him for the moment, the worse he ultimately becomes, just as the child breaking the coal may cause a temporary blaze, but allows the fire all the more quickly, to become smothered in ashes. It would seem that vital processes are much more readily arrested by the accumulation of waste products within the organs of the body than by the want of nutriment of the organs themselves.—*Dr. T. Lander Brunton.*

### DAWN.

The eastern sky is sweet with rosy light,
The glad earth leaps to meet the coming day ;
'Tis June, and dawn ; all Nature's fair array
Shines forth in joyous welcome to the sight.

The call of bird to bird from nest and tree—
The chirp of wakening insect life is heard—
With breath of opening flowers the air is stirred,
The world is full of sweet and melody.

And I, my heart to Love's delight new-born,
Stand midst the roses at the garden gate,
I sing the name of her for whom I wait,
With praise to Him who giveth Love and Dawn.

Loring G. Barry.

